# Metagenomic sequencing for identifying pathogen-specific circulating DNAs and development of diagnostic methods for schistosomiasis

**DOI:** 10.1016/j.isci.2023.107495

**Published:** 2023-07-27

**Authors:** Jingyi Liu, Xiaoxu Wang, Fei Sheng, Bikash R. Giri, Shun Li, Tianqi Xia, Xuxin Li, Guofeng Cheng

**Affiliations:** 1 Shanghai Tenth People’s Hospital, Tongji University School of Medicine, #500 Zhen-nan Road, Shanghai 200331, People’s Republic of China; 2 Shanghai Veterinary Research Institute, Chinese Academy of Agricultural Sciences, Shanghai 200241, People’s Republic of China; 3 School of Biotechnology Jiangsu University of Science and Technology, Zhen Jiang 212100, People’s Republic of China

**Keywords:** Biochemical assay, Genomics, Molecular Genetics

## Abstract

Timely diagnosis of *Schistosoma* infection, particularly in the early stage is crucial for identifying infected hosts and then taking effective control strategies. Here, metagenomic next-generation sequencing was used to identify pathogen-specific circulating DNAs (cDNAs) in the sera/plasma of New Zealand rabbits infected with *S. japonicum*, and the identified cDNAs were validated by PCR and qPCR. Loop-mediated isothermal amplification (LAMP)-based CRISPR-Cas12a and recombinase polymerase amplification-based lateral flow strip (RPA-LF) methods combined with the newly identified cDNA were developed to evaluate the potentials for diagnosing murine and human schistosomiasis. The results indicated that twenty-two cDNAs were identified. The developed LAMP-based CRISPR/Cas12a and RPA-LF methods showed a good potential for diagnosing murine or human schistosomiasis as early as 5 days of post-infection with 5 cercariae infection. In a word, *S. japonicum* specific cDNAs in circulation of infected hosts could be effective biomarkers for detecting *Schistosoma* infection particularly for early stages.

## Highlights

• *S.japonicum* specific cDNAs were identified in infected hosts by metagenomic sequencing
• LAMP-based CRISPR/Cas12a and RPA-LF methods were developed using the newly identified cDNA
• The methods showed a good potential for schistosomiasis diagnosis for early stages

## Graphical abstract

**Figure:**
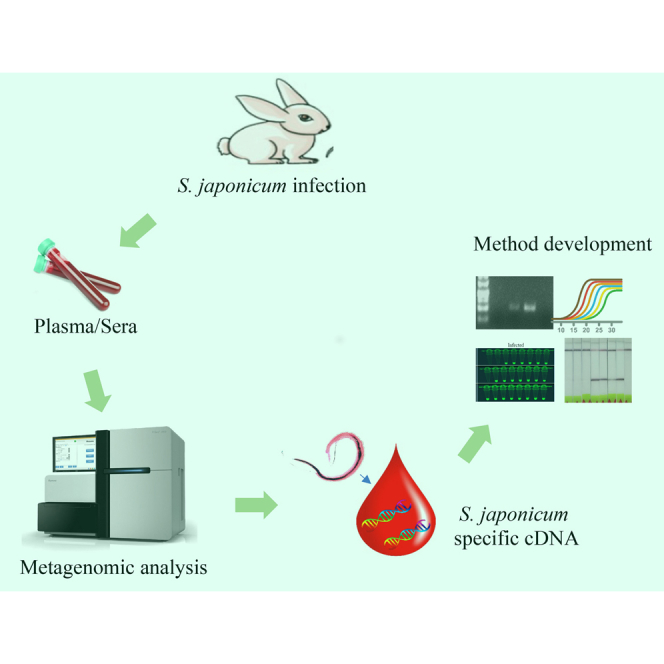

## Introduction

Schistosomiasis is a devastating parasitic disease that affects more than 200 million people worldwide.^1^ Despite remarkable progress in controlling the disease through snails elimination, mass drug administration, and generally combined strategies in China over the past 70 years,^2^ the prevalence of schistosomiasis and the risk of infection still remain an important health problem in some areas.^3^^,^^4^ The widely used method for diagnosing involves microscopy-based detection of parasite eggs in urine (*S. haematobium*) or feces (*S. mansoni* and *S. japonicum*),^5^ which have relatively low-throughput, time-consumption, skilled personnel is required for operation, limiting its diagnostic value in endemic regions.^6^ Additionally, it usually causes an underestimation of the prevalence and infection of this disease due to the results depending on the efficiency of egg-shedding that varies from day to day.^7^ Consequently, sensitive and effective monitoring and/or diagnostic strategies are greatly needed for schistosomiasis control and elimination.^8^

Serological methods for detecting *Schistosoma* infection offers relatively more sensitive and less time-consuming than the microscopy method. However, current serological tests using antigens isolated from eggs or adult worms suffer from lowering specificity and increasing false-positive results.^9^ Alternatively, purified recombinant proteins were suggested to be better antigens for schistosomiasis diagnosis such as saposin-like proteins (SjSAPLP4, SjSAPLP5, and SjSP13),^10^^,^^11^ multidrug-resistant protein 1-encoding gene (SjMRP1),^12^ and Sm-SLP-1^13^ that showed improved specificity. However, the antibodies recognized by these antigens can only be detected in the late stage of *Schistosoma* infection (3–4 weeks after infection),^10^ lack of the capability of detection for early stages. In addition, serological methods are unable to distinguish between ongoing and previous infections.^14^

Recently, nucleic acid-based molecular diagnosis for schistosomiasis was developed and showed high sensitivity and specificity, which are considered to be an attractive diagnostic approach.^15^ Although these methods can detect schistosomiasis in snails, contaminated waters, fecal samples of animals or patients,^16^^,^^17^^,^^18^^,^^19^^,^^20^^,^^21^ the results are largely dependent on the number of eggs or parasites presented in samples used for DNA extraction. Circulating free-cell DNAs (cDNAs), found in blood and other body fluids of mammals have been identified as novel biomarkers for diagnosing different clinical conditions.^22^ Recently, cDNAs derived from some tumors can be used for cancer diagnosis^23^^,^^24^ and cDNAs in maternal plasma can be used for diagnosis of pregnancy and pregnancy-associated complications.^25^ More recently, the usage of parasitic DNA has been successfully validated in the diagnosis of parasites, such as *Plasmodium*,^26^ *Leishmania*,^27^ and *S. mansoni*.^28^ In *S. japonicum*, polymerase chain reaction (PCR), real-time PCR, and loop-mediated isothermal amplification (LAMP) based on molecular detection were also shown to perform relatively high sensitivity in both animal and human schistosomiasis.^29^^,^^30^^,^^31^^,^^32^

Adult *Schistosoma* lives in the blood vessels of final hosts. During the parasitic migration, worm development, and interaction with host immune response, parasitic cells and/or tissues may be turnover and release cDNAs. The cDNA could be distributed throughout the circulation of hosts, which provides an ideal live biomarker for detecting *Schistosoma* infection. Therefore, we performed metagenomic high-throughput sequencing to systematically identify *Schistosoma*-specific cDNAs from the circulation of infected hosts and then developed LAMP-CRISPR/Cas12a and RPA-LF methods for evaluating the potential of identified cDNA for detecting *S. japonicum* infection.

## Results

### Metagenomic next-generation sequencing for identification of pathogen-specific cDNAs in the circulation of rabbits infected with *S. japonicum*

**Figure 1:**
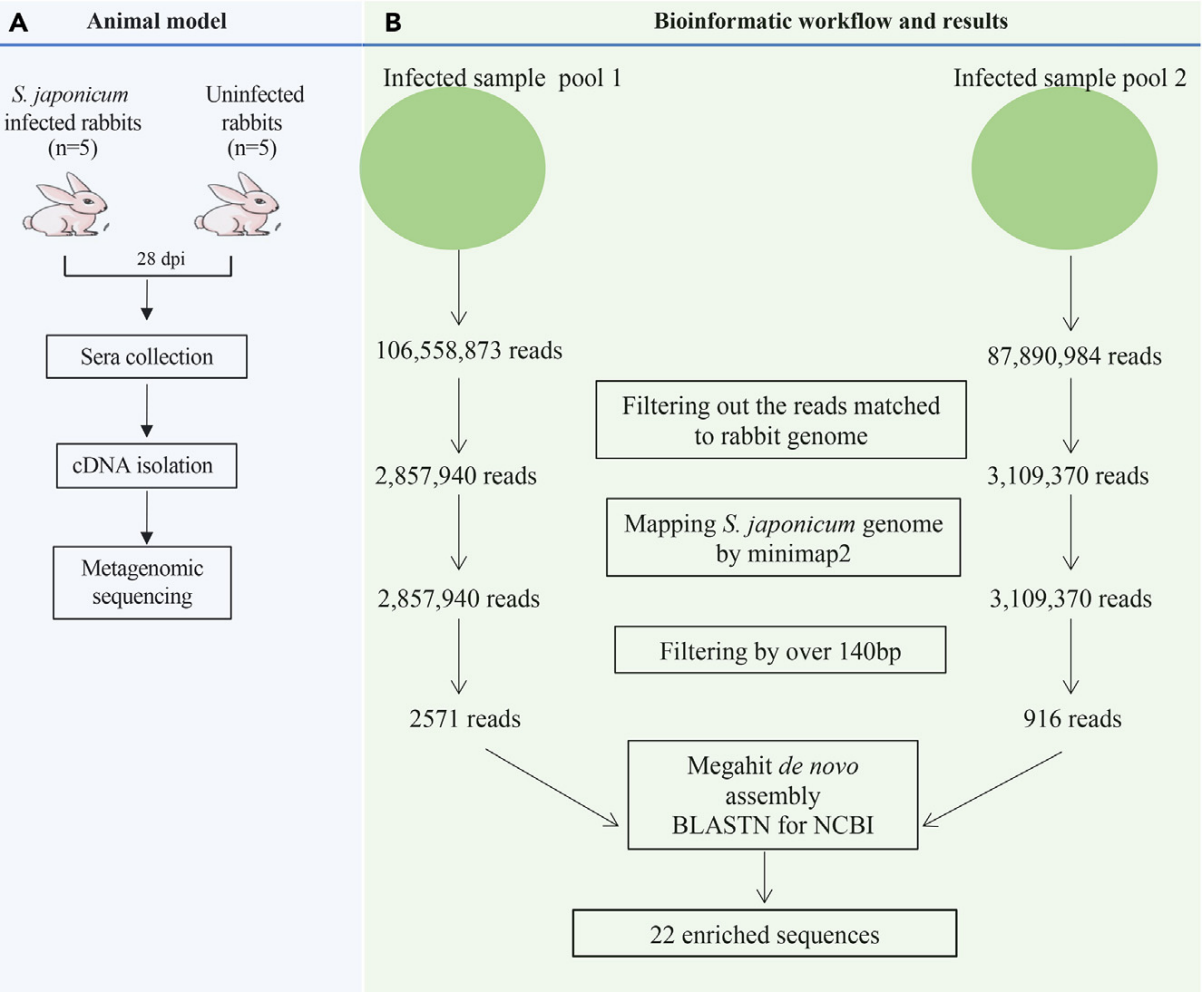
Overview of parasite-specific cDNAs identification by metagenomic sequencing (A) Animal model for metagenomic analysis. (B) Binformatic workflow and results for metagenomic sequencing data. cDNA was isolated from serum samples of rabbits infected with approximately 200–500 cercariae. Following the quality control, the cDNA was fragmented to construct a DNA library that was subsequently sequenced using the Illumina HiSeq platform. The raw data were cleaned and mapped to rabbit genome. After removing reads mapped to the rabbit genome, the other reads were mapped to *S. japoniucm* genome. *S. japonicum* cDNA sequences were obtained by filtering the reads were smaller than 140 bp. Further *de novo* assembly and blast analysis identified twenty-two cDNA sequences.

In total, twenty-two *S. japonicum*-specific cDNAs were identified (Figures 1, S2, and S3, and Table S1). Blast analysis indicated that three cDNAs were matched to SJCHGC08404 protein mRNA (AY812939.1: k141_50, k141_54, and k141_59); four cDNAs were related to non-LTR retrotransposon SjCHGCS19 (FN356221.1: k141_3, 17, 49, and 63); six cDNAs were related to non-LTR retrotransposon SjR2-like sequence (AF412215.1: k141_15, 25, 29, 134; AF412216.1: k141_77 and AF412220.1: k141_143); two cDNAs were related to BAC clones (k141_67 and k141_39); one cDNA was related to non-LTR retrotransposon SjCHGCS20 (FN356222.1: k141_74), LTR retrotransposon SjCHGCS1 (FN356203.1: k141_90), Sj-alpha-1 retrotransposon-like sequence (AF213692.1: k141_125), 28S rRNA gene (Z46504.4: k141_35), SJCHGC09829 protein mRNA (AY915893.1: k141_1), SJCHGC09842 protein mRNA (AY915906.1:k141_56), and SJCHGC09721 protein mRNA (AY915795.1: k141_5), respectively (Table S1).

### Verification of *S. japonicum* cDNAs in sera of infected mice

**Figure 2:**
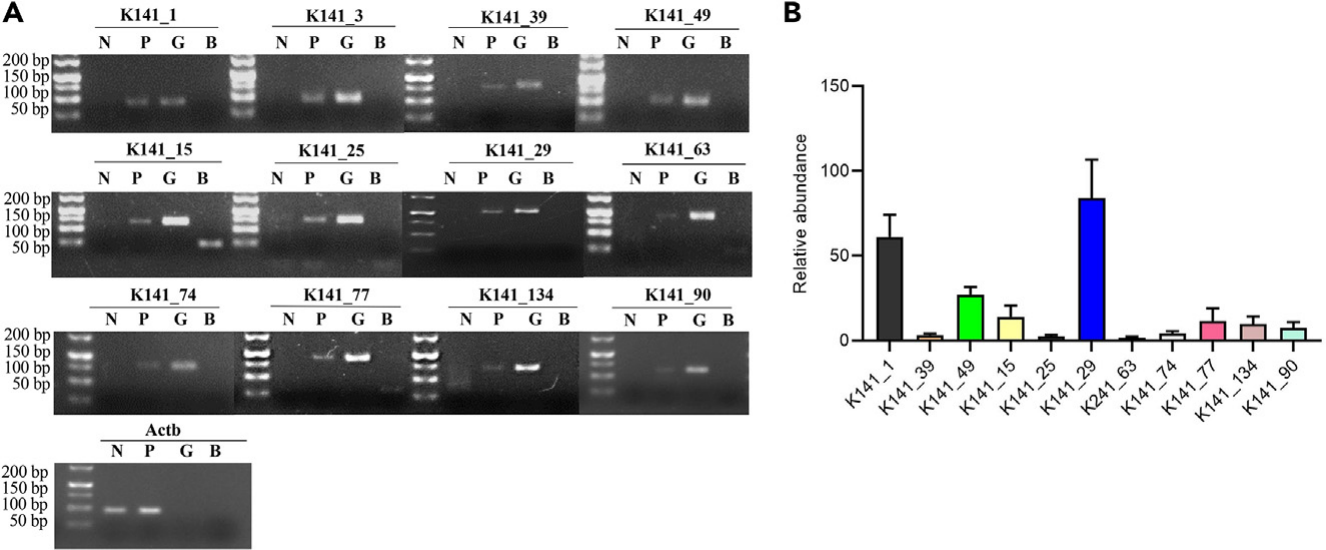
PCR validation of the identified cDNAs in mice infected with *S. japonicum* (A) Agarose gel analysis of PCR products showing 12 out of 22 cDNA sequences were detected in serum samples isolated from mice infected with approximately 40 cercariae. Serum samples isolated from uninfected mice was used as a negative control. Mouse Actb gene was used as an internal control. P means positive sera from infected mice; N means control sera from uninfected mice; G denotes *S. japonicum* genomic DNA isolated from adult worms. b denotes blank. (B) qPCR analysis of the identified cDNAs from murine sera. The pooled cDNA from three individual mice was used as the template for qPCR. Data show representative results and mean and standard errors from triplicate analysis.

To verify the identified cDNAs consistently presented in the circulation of hosts regardless of different species, we designed the PCR primers for 22 identified *S. japonicum* cDNAs and used mice as an animal model to verify the metagenomic next-generation sequencing results. Upon PCR amplification and agarose gel analysis, we found that 12 cDNAs, including K141_1, K141_3, K141_39, K141_49, K141_15, K141_25, K141_29, K141_63, K141_74, K141_77, K141_134, and K141_90 were detected in sera of *S. japonicum*-infected mice while there was no detection in the sera of uninfected animals and blank controls (Figures 2A and S1). To further validate the results, we used qPCR to determine the amount of cDNAs in the circulation of hosts, the results indicated that the cDNAs can be consistently presented and SjR2LS showed the highest amount (Figure 2B). Similar results were also to some extent observed in the experiments with spiked-in control (Figure S4).

### Evaluation of the diagnostic potential of SjR2LS and S20 for *S. japonicum* infection

**Figure 3:**
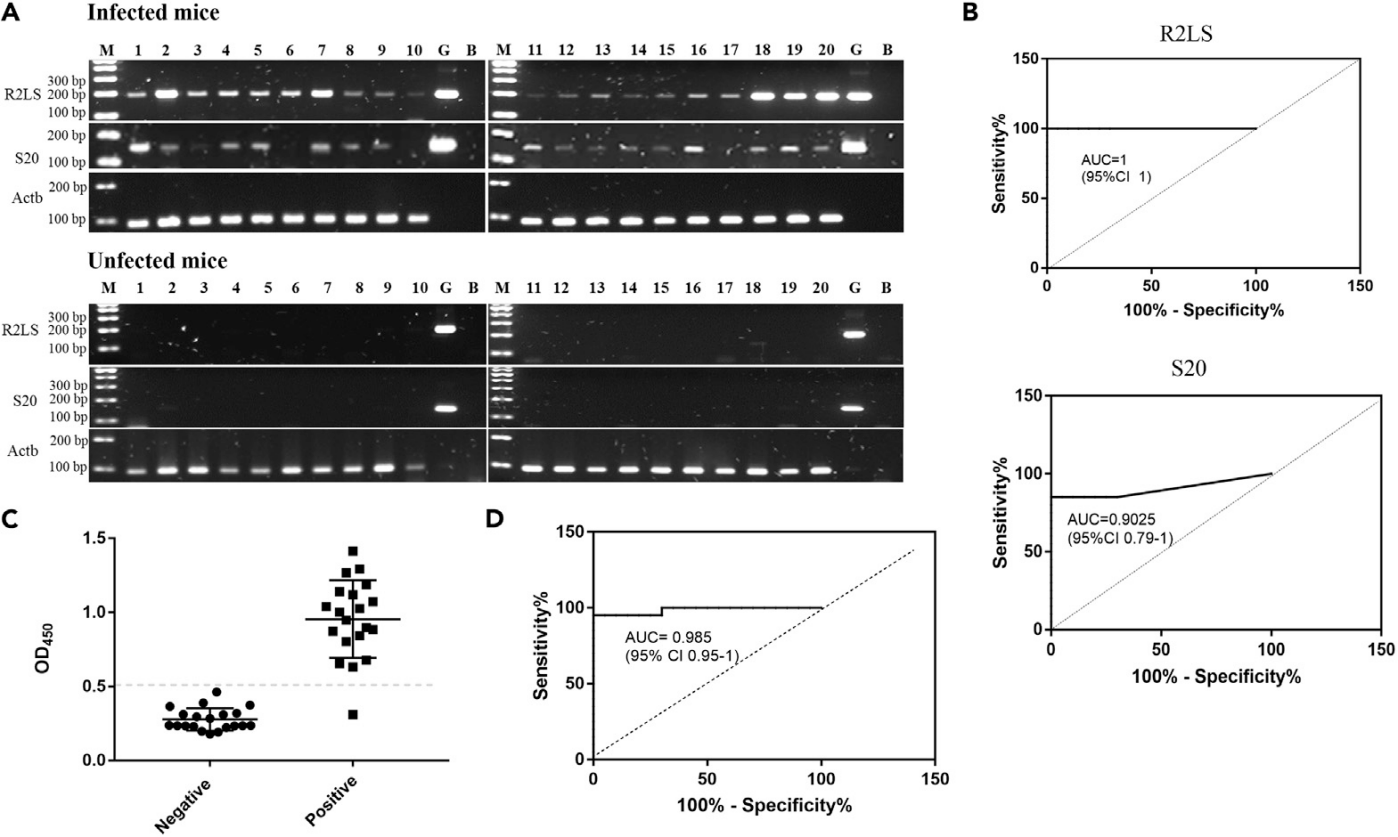
Evaluation of diagnostic potential of SjR2LS and S20 for *S. japonicum* infection (A) PCR amplification and agarose gel analysis of PCR products show that SjR2LS and S20 can be amplified in the majority of mice infected with *S. japonicum*. Mouse Actb gene was used as an internal control. G denotes *S. japonicum* genomic DNA isolated from adult worms; B denotes blank. (B) ROC analysis of the sensitivity of SjR2LS and S20 for detecting *S. japonicum* infection using PCR method based on (A). (C) ELISA analyses of serum samples collected from 20 *S japonicum*-infected mice and 20 healthy mice. One μg egg soluble antigen was used for ELISA analyses. The cut-off is set as 2.1 times the mean OD450 of negative serum samples. (D) ROC analysis of the sensitivity of the ELISA method based on (C).

SjR2-like sequence (SjR2LS, k141_29) and S20 (K141_74) were selected to further investigate the potential of SjR2LS for diagnosing schistosomiasis. Sera from infected mice (n = 20) were subjected to PCR analysis and ELISA analysis. SjR2LS was detected in all of sera samples collected from mice infected with *S. japonicum* while relatively less number of infected mice was observed when applying S20 (Figures 3A and S1). ImageJ analysis of Figure 3A for performing ROC analysis indicated the diagnostic value of SjR2LS (AUC = 1 for SjR2LS) is better than that of S20 (AUC = 0.9025 for S20) (Figure 3B). Sera collected from mice at 30 days of post *S. japonicum* infection as well as uninfected mice were analyzed by using ELISA method. The results indicated the diagnostic accuracy of ELISA (AUC = 0.985) (Figure 3C) is less sensitive than that of PCR-based SjR2LS (Figure 3D).

### PCR-based SjR2LS method is more sensitive than ELISA method

**Figure 4:**
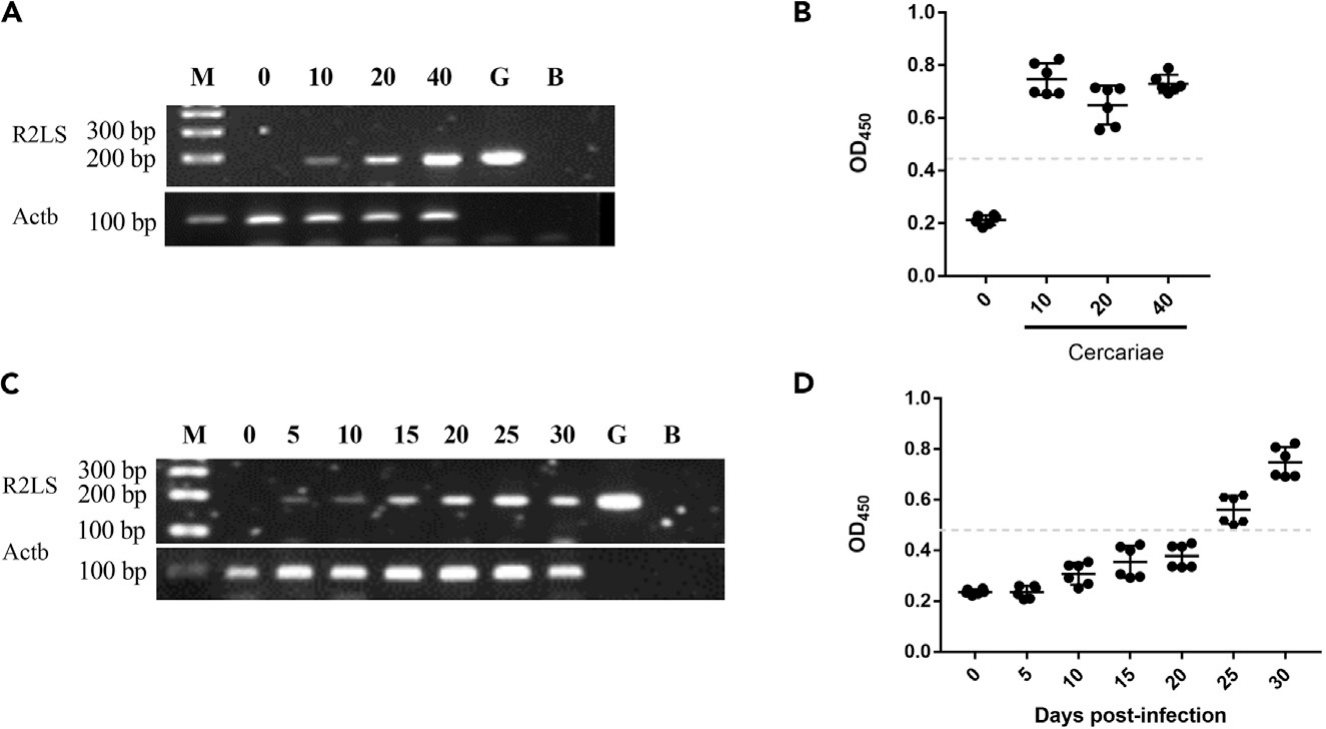
Evaluation of sensitivity of SjR2LS-based PCR method for detecting *S. japonicum* infection at early stage (A) PCR analyses shows that SjR2LS can be amplified in all serum samples from mice infected with 10, 20 and 40 cercariae at 30 days of post-infection. Mouse Actb gene was used as an internal control. (B) ELISA analyses showing that *S. japonicum* infection can be detected in mice infected with 10, 20 and 40 cercariae at 30 days of post-infection (n = 6). (C) PCR analyses shows that SjR2LS can be amplified in serum samples from infected mice burden with 5 worms at 5, 10, 15, 20, 25 and 30 days of post-infection. Mouse Actb gene was used as an internal control. (D) ELISA analyses shows that *S. japonicum* infection can be detected in infected mice burden with 5 worms at 25 and 30 days of post-infection (n = 6).

To further confirm the sensitivity of SjR2LS for detecting *S. japonicum* infection, twenty-four mice were randomly divided into four groups including one uninfected group and three infected groups with different 10, 20, 40 cercariae inoculations, respectively. Sera were collected from infected mice at 5, 10, 15, 20, 25, and 30 days of post-infection, respectively. Worm burden in each mouse was also observed at 30 days of post-infection. SjR2LS was determined by PCR from sera collected from mice at 30 days of post-infection. The results indicated that SjR2LS was able to be detected in the cDNAs isolated from sera samples of mice that perfused to collect at least 5–7 worms (Figures 4A and S1) while similar results were also observed using ELISA method (Figure 4B). Importantly, we observed that the detectable capacity using SjR2LS in infected mice with 5 worms can be observed as early as 5 days of post-infection (Figure 4C) while ELISA method was showed the credible detection at least at 25 days of post-infection (Figure 4D). These results indicated that detection of *S. japonicum* infection using SjR2LS-based PCR is more sensitive than that of ELISA method for early stage.

### LAMP-based CRISPR/Cas12a method for detecting *S. Japonicum* infection

**Figure 5:**
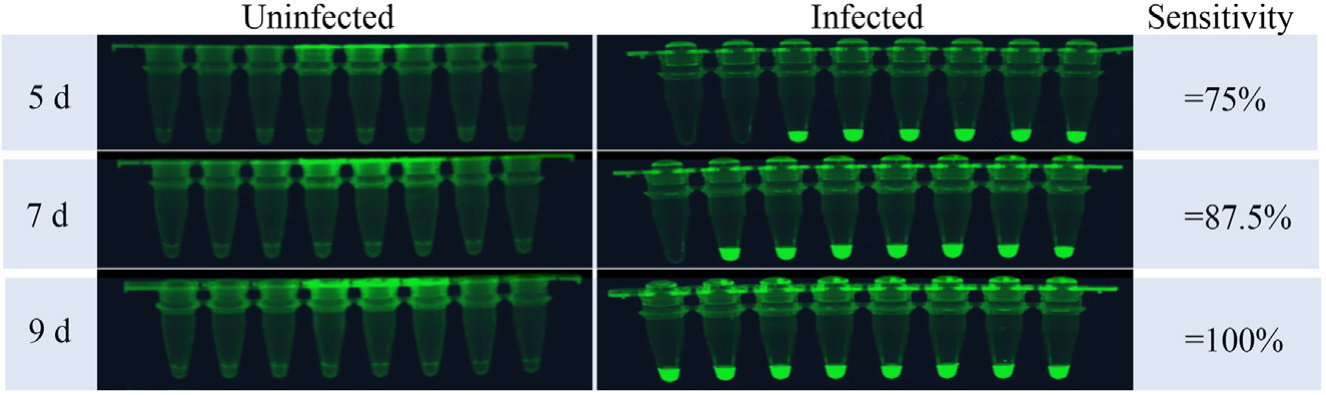
Evaluation of LAMP-based CRISPR/Cas12a method for SjR2LS for detecting *S. japonicum* infection Sixteen mice were randomly divided into two groups (infection and control) and each mouse in the infection group was infected with 5 cercariae. The sera from infection groups and controls were collected at indicated times and an LAMP-based CRISPR/Cas12a assay was performed as described in the STAR methods section.

Upon the optimized conditions for LAMP assay as well as the identification of digest activity for CRISPR/Cas12a (Figures S1 and S5), we developed a LAMP-based CRISPR/Cas12a combined with the fluorescent reporter to increase the sensitivity for detection. Upon analyses of plasmas collected from the uninfected control and infected mice at different days of post-infection (5 days, 7 days, and 9days), we found that the percentage of detection are 75%, 87.5%, and 100%, respectively (Figures 5 and S1). The results indicated that the LAMP-based CRISPR/Cas12a method can to some extent detect *S. japonicum* infection as early as 5 days of post-infections.

### RPA-LF method for detecting *S. japonicum* infection

**Figure 6:**
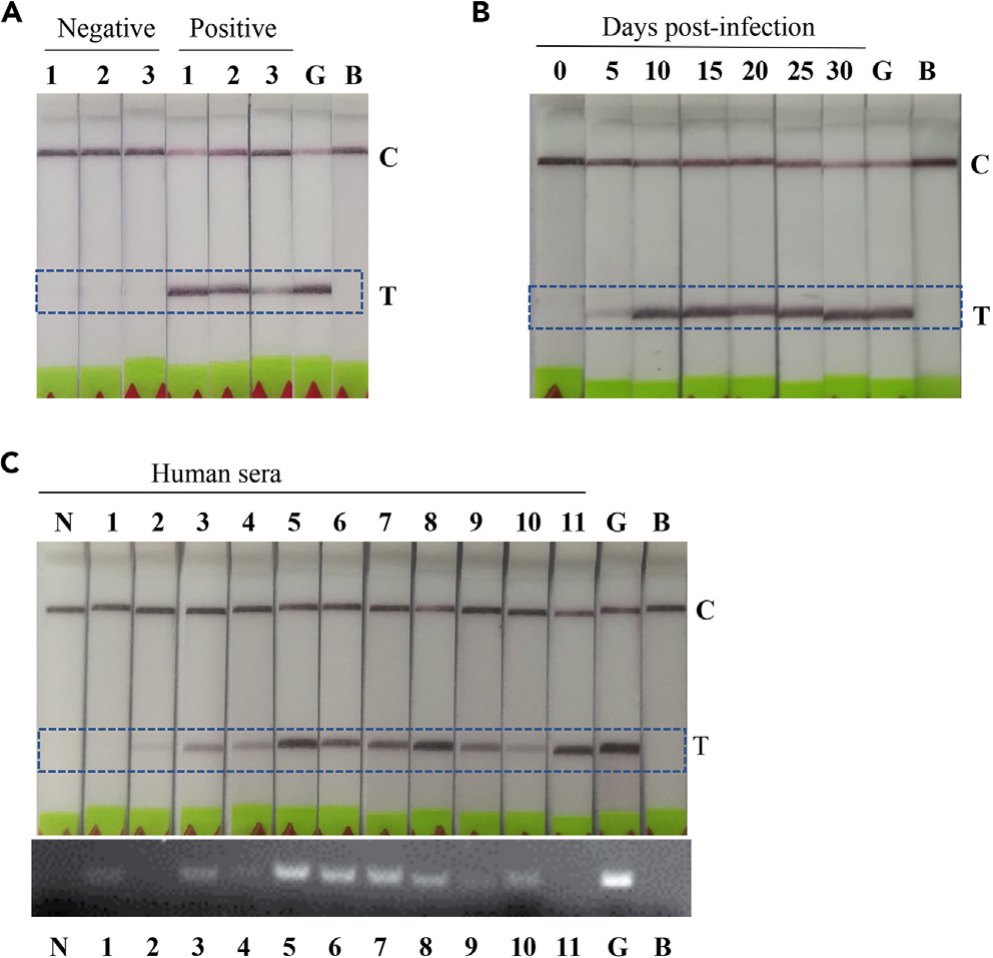
Development of LF-RPA approach for detecting *S. japonicum* infection (A) Evaluation of SjR2LS-based LF-RPA approach for detecting *S. japonicum* infection. Forty mice were randomly divided into two groups (infection and control) and each mouse in the infected group was infected with 40 cercariae and sera were collected at 20 days of post-infection. Three infected and control serum samples were randomly selected for LF-RPA analysis. (B) Evaluation of detectable capacity of SjR2LS-based LF-RPA method. Twelve mice were randomly divided into two groups (infection and control) and each mouse in the infected group was infected with 5–10 cercariae. The sera from infected groups and controls were collected at indicated times as described in the method section and were further analyzed by LF-RPA. (C) Evaluation of diagnostic capacity of SjR2LS-based LF-RPA for human sera collected from human schistosomiasis. LF-RPA results showed that SjR2LS can be amplified in 10 out of 11 serum samples from human schistosomiasis japonica, which is more sensitive than the PCR method that detects 9 out of 11 samples. T indicated test samples; C indicated control for assay.

Alternatively, we further developed a SjR2LS-based rapid polymerase amplification combined with a lateral flow strip (RPA-LF) assay. Firstly, positive serum samples isolated from mice infected with *S. japonicum* at 20 days of post-infection and uninfected serum samples were evaluated using the developed RPA-LF assay. As shown in Figure 6A, SjR2LS can be amplified by LF-RPA in the sera of infected mice, while no signal was observed in sera isolated from uninfected mice. As to the sensitivity of RPA-LF, we noted that SjR2LS was able to be detected in mice infected with 5 cercariae as early as 5 days of post-infection (Figures 6B and S1). Furthermore, we evaluated the diagnostic performance of the SjR2LS-based RPA-LF for diagnosing schistosomiasis in human patients. As shown in Figure 6C, the positive signal for SjR2LS can be observed in 10 out of 11 serum samples of human schistosomiasis, which is more sensitive than SjR2LS-based PCR method, that detect 9 out of 11.

## Discussion

Current methods for schistosomiasis diagnosis usually suffer from time-consuming, lack of sensitivity, or less capability of detection for early infection. Consequently, there is a great need to develop an effective method showing live parasites with high sensitivity for detecting *Schistosoma* infection, particularly for the early stage.^33^ Since *Schistosoma* live in the blood vessels of the host, pathogen-specific cDNAs in the circulation of the infected hosts may provide a unique advantage for developing live-infection biomarkers for diagnosing schistosomiasis. Currently, nucleic acid-based methods such as PCR, real-time PCR, LAMP, and RPA have been successfully developed and implicated in diagnosing schistosomiasis.^28^^,^^32^ The sensitivity and specificity of these methods are affected by various factors; one of the key factors is biomarkers. Several DNA biomarkers have been evaluated for schistosomiasis diagnosis including Dra1 sequence of *S. haematobium* (GenBank: DQ157698.1),^34^ a 121 bp tandem repeated DNA sequence of *S. mansoni*,^28^^,^^35^ and SjCHGCS19 and SjR2 of *S. japonicum*.^29^^,^^30^^,^^36^ However, the effective biomarker for *S. japonicum* infection is still needed.

In the present study, we systematically identified cDNAs from plasma/sera of rabbits infected with *S. japonicum,* resulting in 22 cDNAs that matched to *S. japonicum* genome. The resulted cDNAs are not reported previously. Although a couple of cDNAs were shown to match some GenBank Gene IDs, such as SjCHGCS19 (GenBank: FN356221.1) that have been evaluated their diagnostic potential in a previous study.^30^ Our findings indicated that different regions related to the genes are matched to the identified cDNA. Importantly, we identified six SjR2-like cDNAs, including AF412215.1 (k141_15, 25, 29, 134), AF412216.1 (k141_77), and AF412220.1 (k141_143), while SjR2 (GenBank: AF412221.1) detected in sera samples of animals infected with *S. japonicum*^29^^,^^30^^,^^36^ was not identified in our present study. We noted there are different regions presented as cDNA for one gene, suggesting that cDNAs may be released in region-dependent manners.

Additionally, we further validate the identified cDNAs to be detectable in *S. japonicum*-infected mice sera, confirming that 12 out of 22 can serve as biomarkers for diagnosing murine schistosomiasis. Further investigation of SjR2LS indicated that the detection rate of *S. japonicum* infection is 100% in mice infected with *S. japonicum*, which is more sensitive than the ELISA method. LAMP and RPA are more attractive for field application because the amplification can be done rapidly at a constant temperature and naked eyes can visualize the results. We combined LAMP with CRISPR/Cas12a to improve the sensitivity by using fluorescent labeled probes. The results indicated that LAMP-CRISPR/Cas12a-based method could detect *S. japonicum* infection as early as 5 days with 5 cercariae. Additionally, RPA has more advantages, such as being done at a significantly low temperature (37°C–42°C) and a rapid time (within 20 min). In previous studies, real-time RPA and LF-RPA assays targeting the SjR2 (AF412221.1) have been investigated to detect *S. japonicum* infection in the fecal or serum samples of patients.^37^^,^^38^ Here, we documented an alternative SjR2LS and further developed two methods, including LAMP-CRISPR/Cas12a and LF-RPA. It was shown that SjR2LS can be amplified by LF-RPA assay in cDNAs isolated from serum samples of infected mice within 15 min. Furthermore, higher sensitivity of the established LF-RPA method was observed than the PCR method when implicated in diagnosing the serum samples from persons infected with *S. japonicum*.

In summary, metagenomic next-generation sequencing for identification of parasite-specific cDNAs associated with *S. japonicum* infection was carried out in the present study and twenty-two cDNAs were found and validated in different animal models. We also developed LAMP-CRISPR/Cas12a and LF-RPA methods combined with newly identified cDNA and applied to *S. japonicum*-infected animal and human samples successfully. These findings provided a resource for establishing sensitive molecular approaches for diagnosing schistosomiasis. The developed LAMP-CRISPR/Cas12a and LF-RPA assays may represent an alternative method for detecting *Schistosoma* infection, particularly for the early stage.

### Limitations of the study

This study presents the identification of *S. japonicum*-specific cDNAs in plasma/sera rabbits and verification of some of these identified cDNAs by PCR, qPCR, and the developed LAMP-CRISPR/Cas12a and LF-RPA methods. However, some issues still require further investigation. Firstly, it is unclear about the relativity between the abundance of identified *S. japonicum*-specific cDNAs and different hosts. Secondly, due to the availability of human sera for schistosomiasis patients, we only evaluated 11 patients using the developed LF_RPA method in the present study. It is necessary to increase sample size to further validate the developed methods.

## STAR★Methods

### Key resources table

**Table:** 

REAGENT or RESOURCE	SOURCE	IDENTIFIER
**Parasites**		

*S. japonicum* cercariae	National Institute of Parasitic Disease, Chinese Center for Disease Control and Prevention	N/A

**Biological samples**		

Animal schistosomiasis serum samples	Rabbits, mice	N/A
Human schistosomiasis serum samples	National Institute of Parasitic Disease, Chinese Center for Disease Control and Prevention	N/A

**Chemicals, peptides, and recombinant proteins**		

LbCas12a (Cpf1) Nuclease	TOLOBIO	Cat# 32108-03
Bst DNA Polymerase Large Fragment	Vazyme	Cat# P701-01
3,3′,5,5′-tetramethyl benzidine dihydrochloride (TMB)	TIANGEN	Cat# PA107
BSA	SIGMA	Cat# A4737
HRP conjugated goat anti-mouse IgG	CWBIO	Cat# CW0102

**Critical commercial assays**		

QIAamp Circulating Nucleic Acid Kit	Qiagen	Cat# 55114
TSINGKE®MasterqPCR Mix	Tsingke Biotechnology	Cat# TSE201
DreamTaq Green PCR Master Mix	ThermoFisher Scientific	Cat# K1081
MEGAscript™ T7 Transcription Kit	ThermoFisher Scientific	Cat# AM1333
TwistAmp nfo kit	TwistDX	Cat# TANFO02KIT
Milenia Genline HybriDetect 1	TwistDX	Cat# MILENIA01
Qubit™ 1X dsDNA HS Assay Kit	ThermoFisher Scientific	Cat# Q33230
TruSeq DNA LT Sample Prep Kit	Illumine	Cat# FC-121-2001
Agencourt AMPure XP 60 mL kit	Beckman Coulter Genomics	Cat# A63881
Bradford assay	ThermoFisher Scientific	Cat# 23236

**Deposited data**		

Metagenomic raw data	This paper	NCBI Sequence Read Archive (SUB13513518)

**Oligonucleotides**		

Primers for PCR and qPCR, see Tables S2 and S3	Sangon	N/A
Primers for LAMP, see Table S4	Sangon	N/A
Primers and probes for RPA, see Table S5	Sangon	N/A

**Software and algorithms**		

GraphPad Prism	https://www.graphpad.com/	GraphPad
FastQC	https://www.bioinformatics.babraham.ac.uk/projects/fastqc/	FastQC
Cutadapt (v1.2.1)	https://cutadapt.readthedocs.io/	Cutadapt
BWA	http://bio-bwa.sourceforge.net/	Burrows-Wheeler-Alignment Tool
Minimap2 (2.17-r954-dirty)	https://github.com/lh3/minimap2#general	Minimap2
Megahit (v1.1.2)	https://github.com/voutcn/megahit	Megahit
Blastn (2.2.31+)	https://ftp.ncbi.nlm.nih.gov/blast/	Blastn

### Resource availability

#### Lead contact

Further information and requests for resources and reagents should be directed to and will be fulfilled by the lead contact, Guofeng Cheng (cheng_guofeng@foxmail.com or chengguofeng@tongji.edu.cn).

#### Materials availability

This study did not generate new unique reagents.

### Experimental model and study participant details

#### Animal experiments

The life cycle of *S. japonicum* (Anhui isolate) were maintained in New Zealand rabbits and Kunming mice and *Oncomelania hupensis* was obtained from the National Institute of Parasitic Disease, Chinese Center for Disease Control and Prevention (Shanghai, China). New Zealand rabbits with a weight of 2.5–3 Kg (male, for each group, n = 5) were infected with approximately 200–500 cercariae via abdominal skin penetration. For mice experiment I, two groups of eight-week-old male Kunming mice (for each group, n = 20) were used. One group of mice were challenged with approximately 40 cercariae while another group served as non-infected control. For mice experiment II, four groups of eight-week-old male mice (for each group, n = 6) were used. One group of non-infected mice were served as control, while the other three groups were challenged with 10, 20 and 40 cercariae, respectively. For mice experiment III, two groups of eight-week-old male Kunming mice (for each group, n = 8) were used and each mouse was challenged with 5 cercariae in infected group while another group served as non-infected control. For mice experiment Ⅳ, two groups of eight-week-old male Kunming mice (for each group, n = 6) were used and each mouse was challenged with 5–10 cercariae in infected group while another group served as non-infected control (Figure S1).

The study was approved by Shanghai Laboratory Animal Management Committee and the Animal Care and Use Committee of Shanghai Veterinary Research Institute, Chinese Academy of Agricultural Sciences (Permit number: SYXK 2016-0010) and Internal Review Panel of Laboratory Animal Research Center, Tongji University.

#### Serum/plasma samples

For rabbit sera, approximately 20 mL of blood was collected from infected rabbits at 28 days of post-infection. For mice experiment I, the mice were sacrificed at 30 days of post-infection, serum was collected from individual mouse and worm burden from each mouse were counted simultaneously. For mice experiment II, serum samples were collected at 5, 10, 15, 20, 25 and 30 days of post-infection, respectively. Then, mice were sacrificed at 30 days of post-infection to assess worm burden. For mice experiment III, plasma samples were collected at 5, 7 and 9 days of post-infection, respectively. For mice experiment Ⅳ, serum samples were collected on at 5, 10, 15, 20, 25, and 30 days of post-infection, respectively. Human serum samples were provided kindly by Profs Cao Jianping and Yujuan Shen from the National Institute of Parasitic Disease, Chinese Center for Disease Control and Prevention (Shanghai, China).

### Methods details

#### cDNA extraction and metagenomic high-throughput sequencing

cDNA was extracted using a circulating nucleic acid extraction kit (Qiagen, Germany) according to the manufacturer’s instructions. Briefly, 10 mL of rabbit serum were mixed with 1 mL proteinase K and 8 mL buffer ACL. Upon incubation, 18 mL of buffer ACB was added. Then, the mixture was transferred to the QIAamp mini column and followed by several washes. The mini column was dried at 56°C for 10 min and eluted by 35 μL AVE buffer. For the mouse and human serum samples, 90–400 μL of serum from each sample was used to extract cDNA as described previously. The quality and quantity of the isolated cDNA were assessed by a NanoDrop ND-1000 spectrophotometer (Thermo Fisher Scientific, Waltham, MA, USA). The cDNA was processed to construct the library by a TruSeq DNA Nano High Throughput Library Preparation Kit (Illumina, San Diego, CA, USA) and sequence using the Illumina Hiseq (Illumina, San Diego, CA, USA).

#### Bioinformatics analysis

Raw data were first processed using Cutadapt (v1.2.1) to obtain clean reads. Then, the clean reads were mapped to rabbit genome using BWA (http://bio-bwa.sourceforge.net/).^39^ Following the removal of sequences that matched to rabbit genome, *S. japonicum* cDNA sequences were obtained by mapping the leftover reads to *S. japonicum* genome using minimap2 (2.17-r954-dirty). The data were further filtered by removing the reads smaller than 140 bp. After that, the SjcDNA sequences were obtained by *de novo* assembling the reads using megahit (v1.1.2) (https://hku-bal.github.io/megabox/) and blast the contigs in NCBI database (ftp://ftp.ncbi.nih.gov/blast/db/, v2016-6-19) (Figure S2).

#### PCR and qPCR analysis of identified cDNAs

PCR was performed with the listed primers (Table S2) in a volume of 25 μL, including 12.5 μL 2x DreamTaq PCR master mix (Invitrogen, USA), 0.5 μL of each primer (10 μM), 2 μL of isolated cDNA, added nuclease-free H_2_O to 25 μL. PCR products were analyzed on 2% agarose gel. A murine beta actin gene (Actb, GenBank NM_007393.5) was used as internal controls. qPCR was performed using SYBR green I Master mix (Tsingke Biotechnology) in ABI7500 (Thermofisher Scientific, USA). The 20 μL PCR sample contained 2 μL of isolated cDNA product (1:5 dilution), 10 μL 2×SYBR green I Master mix, 1.6 μL Primer mix, and 6.4 μL H_2_O. All reactions were run in at least triplicate. We also spiked in the plasmid as internal control (0.01 ng/μL) to perform qPCR. We used the 2^−ΔCt^ method to calculate relative expression. The primers used for qPCR are listed in Table S3.

#### LAMP-based CRISPR/Cas12a

The LAMP reaction was performed with the listed primers (Table S4) in a volume of 25 μL containing 2.5 μL 10× ThermoPol Buffer (2.5 μL), 1 μL MgSO4 (2–12 mM), 3.5 μL 10mM dNTP Mix (10 mM), 4 μL 10 μM FIP/BIP Primers (1.6 μM), 0.5 μL 10 μM F3/B3 Primers (0.2 μM), LF/LB Primers (0.8 μM), Bst DNA Polymerase Large Fragment (10 U), Calcein (25 μM), 2 μL of isolated cDNA, added nuclease-free H_2_O to 25 μL. The reaction was incubated at 65°C for 30 min1 h and then inactivated at 85°C for 5 min.

For CRISPR/Cas12a, crRNA was obtained using the MEGAscript T7 Transcription Kit (Thermofisher Scientific) using the below primers and annealed *in vitro*. The *in vitro* transcribed products were purified and the purified RNA was used as crRNA.

Next, the product was added CRISPR/Cas12a. Briefly, 20 μL reactions include 2 μL 10 x TOLO Buffer, 2μL crRNA (2.5 μM), 1 μL Cas12a Nuclease (1μM), 1 μL LAMP product, 1 μL ssDNA (500nM, 5ʹ-FAM-ssDNA-BHQ1-3ʹ), and added nuclease-free H_2_O to 20 μL. The mixture was incubated at 37°C for 30 min and then inactivated at 85°C for 10 min. The results were observed with UV light and a SpectraMax M5 microplate reader (Molecular Devices, CA, USA).

#### ELISA

ELISA was performed in a 96 well plate (Costar, USA) coated with 100 μL of egg antigens (10 μg/mL) with slight modification.^40^ The 96-well plate (Costar, USA) was coated with 100 μL of SEA (10 μg/mL) in carbonate-bicarbonate buffer (pH 9.0) per well at 4°C overnight. Then, the plate was washed with PBS containing 0.05% Tween 20 (PBST) for three times and blocked with 100 μL 1% BSA in PBST at 37°C for 1 h. The plate was further washed with PBST and incubated with 100 μL serum diluted at 1:100 at 37°C for 1 h. The plate was washed with PBST and incubated with 100 μL horseradish peroxidase-conjugated goat anti-mouse IgG antibody diluted at 1:5000 at 37°C for 30 min. Following three washes, 100 μL 3,3′,5,5′-tetramethyl benzidine dihydrochloride (TMB) was added to each well and incubated for 10 min. Then, 2M sulfuric acid was added to stop the reactions and the absorbance was determined using a microplate reader (BioTek, USA) at 450 nm.

#### LF-RPA

Primers and probes specific to the k141_29 were designed according to the TwistDX instructions and further evaluated by TwistAmp nfo kit (TwiatDx, UK). An internal probe with a FAM, THF and C3 spacer were designed and a biotin was added at the 5ʹ end of the reverse primer (Invitrogen). The primers and probes were listed in Table S5. RPA reaction was carried out using TwistAmp nfo kit (TwistDX, UK) following the manufacturer’s instructions with 50 μL reaction mixture. To analyze the results, 2 μL RPA reaction mixture was mixed with 98 μL dipstick assay buffer and 10 μL of the diluted sample was transferred to the sample pad of a Genline Hybridetect-1 lateral flow strip (Milenia Biotec, Germany). The strip was vertically placed in 200 μL of PBST buffer and incubated for 5 min at room temperature.

### Quantification and statistical analysis

The ELISA results were presented as the mean ± SEM (standard error of the mean). The cutoff value was computed as 2.1 times of the mean absorbance values of the *S. japonicum* negative sera. Receiver-operating characteristic (ROC) curves and area under the curve (AUC) values were generated for the cDNA using the GraphPad Prism 7.0. Details for each figure are included in the Figure Legends.

## Data Availability

• The raw sequence data reported in this paper have been deposited in the NCBI SRA database: PRJNA982300. • This paper does not report original code. • The datasets are publicly available. Any additional information required to reanalyze the data reported in this paper is available from the lead contact upon request.
