# Supplementary material for: Metagenomic sequencing for identifying pathogen-specific circulating DNAs and development of diagnostic methods for schistosomiasis
Source: iScience. 2023 Jul 27;26(9):107495. doi: 10.1016/j.isci.2023.107495 (PMC10457526; doi:10.1016/j.isci.2023.107495)
Supplement: Document S1. Figures S1–S5 and Tables S1–S5 [file mmc1.pdf]

iScience, Volume *26*

Supplemental information

Metagenomic sequencing for identifying pathogen-specific circulating DNAs and development of diagnostic methods for schistosomiasis

Jingyi Liu, Xiaoxu Wang, Fei Sheng, Bikash R. Giri, Shun Li, Tianqi Xia, Xuxin Li, and Guofeng Cheng

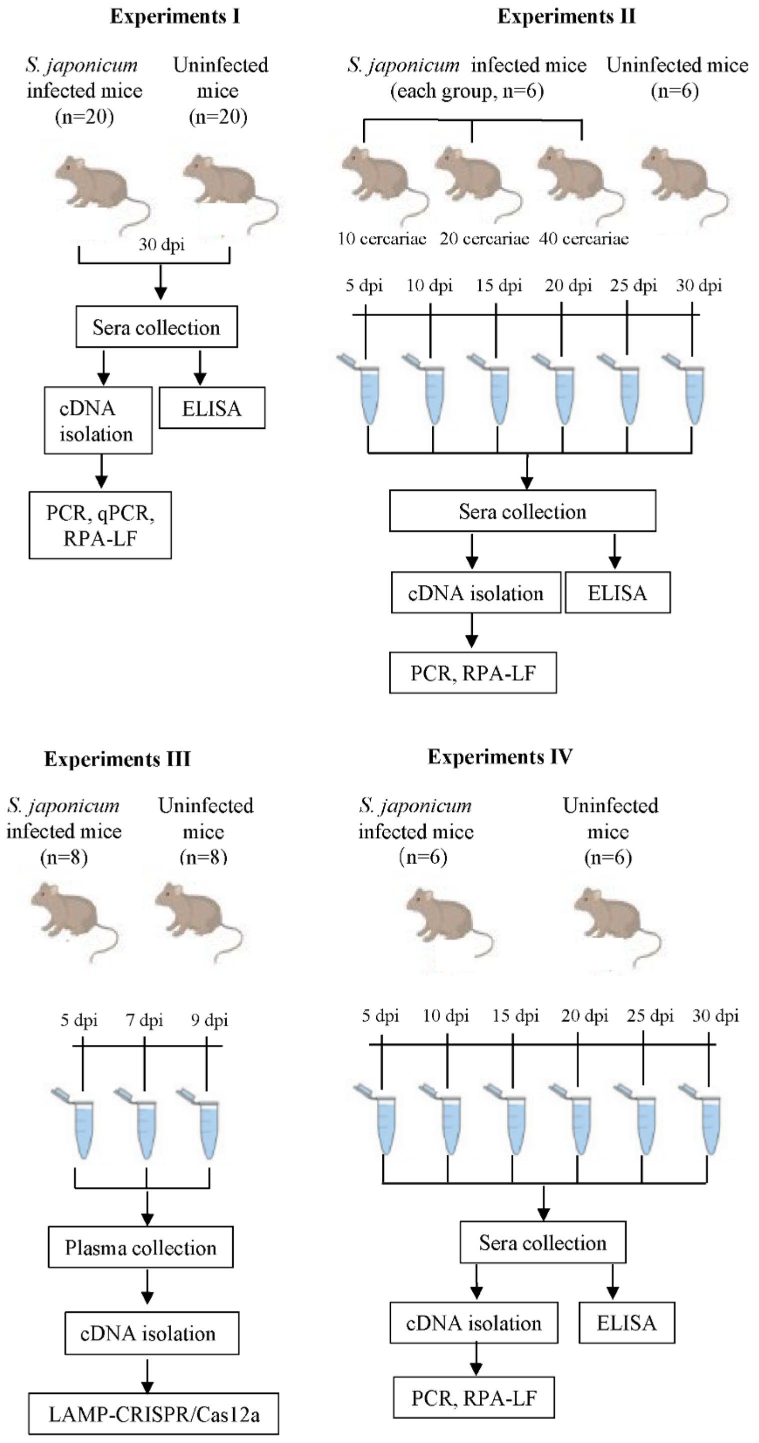

Figure S1, related to Figure 2A, 3A, 4A, 5, and 6B. Overall of animal experiments in the present study.

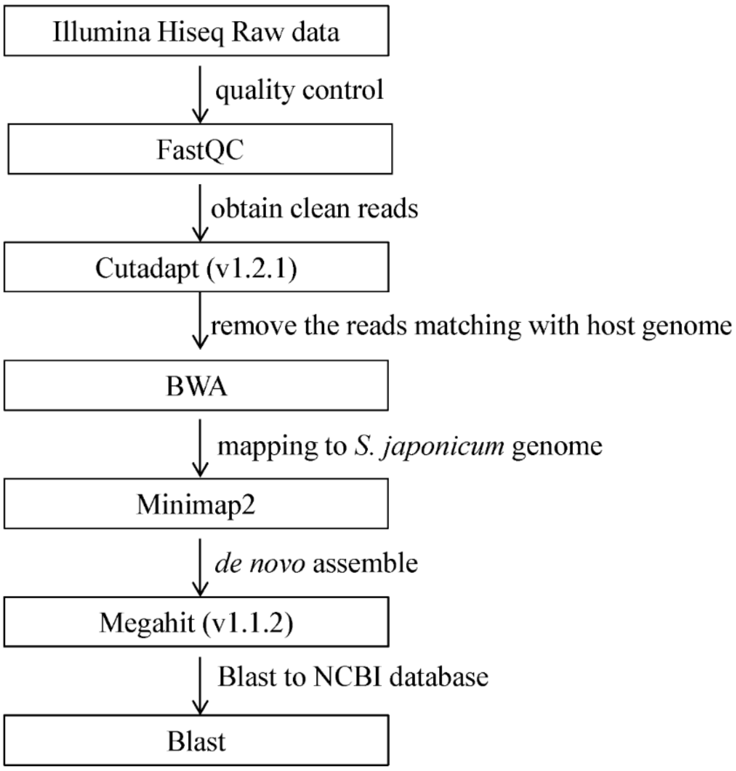

Figure S2, related to Figure 1. Workflow of bioinformatic analysis of metagenomic data.

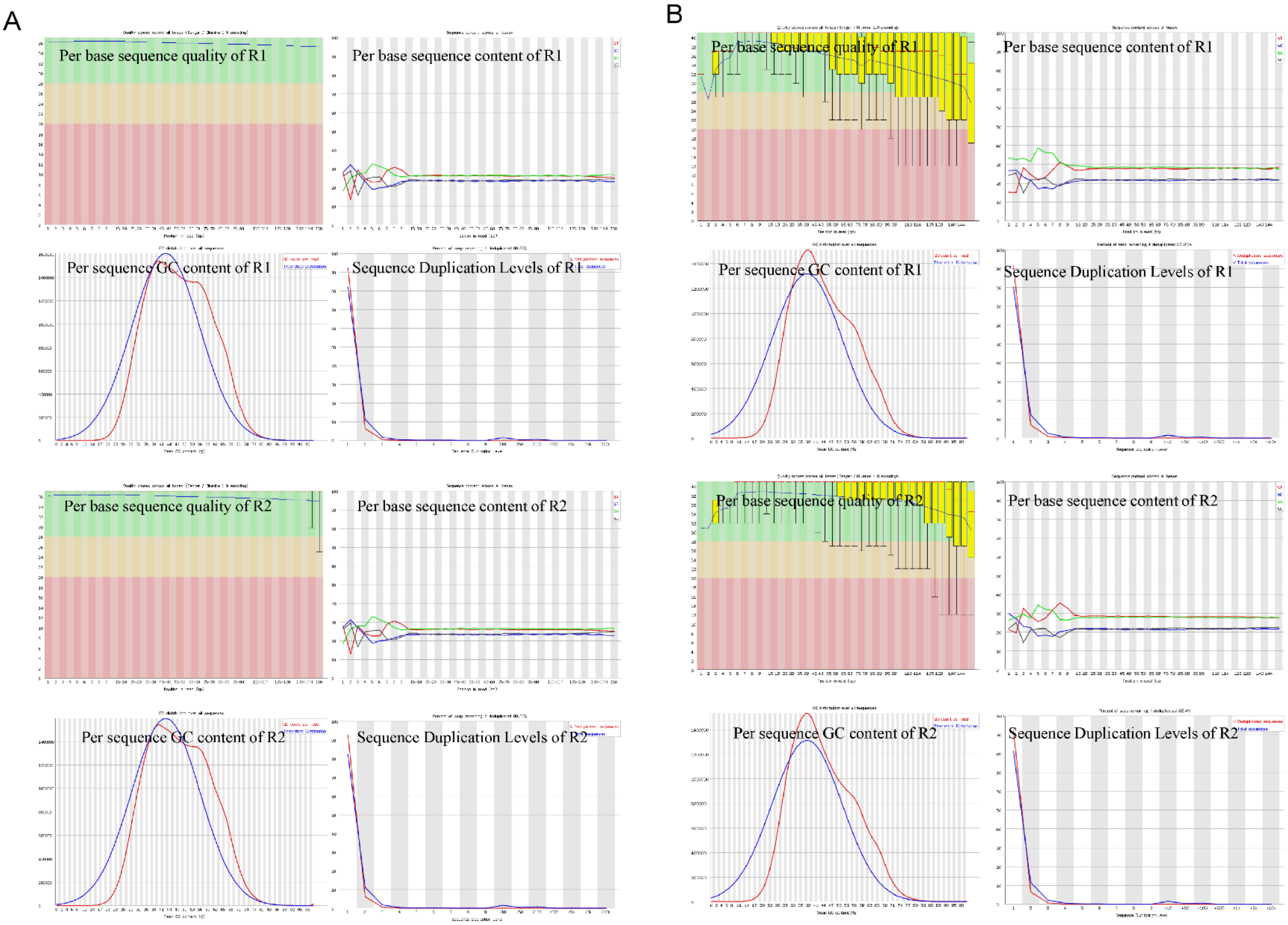

Figure S3, related to Figure 1. FastQC analysis of Illumina Hiseq Raw data.

A. Infected sample pool 1

B. Infected sample pool 2

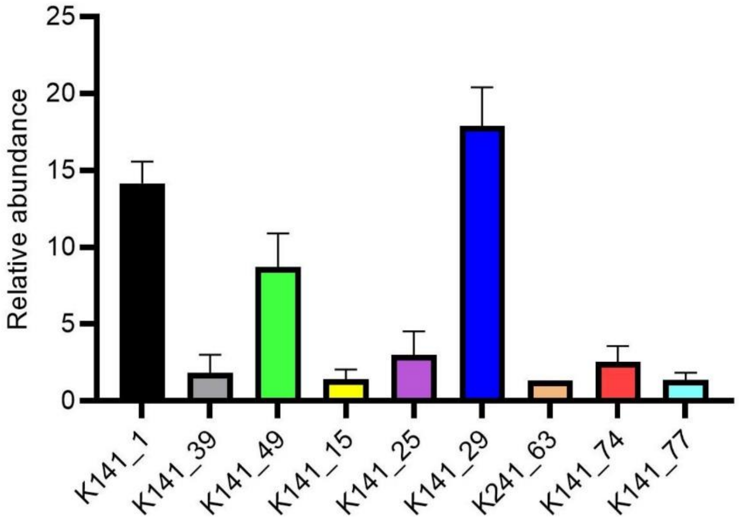

Figure S4, related to Figure 2B. qPCR analysis of the identified cDNAs from murine sera using spiked-in plasmids as the controls. The pooled cDNA from three individual mice with spiked in plasmid was used as the template for qPCR. Data show representative results and mean and standard errors from triplicate analysis.

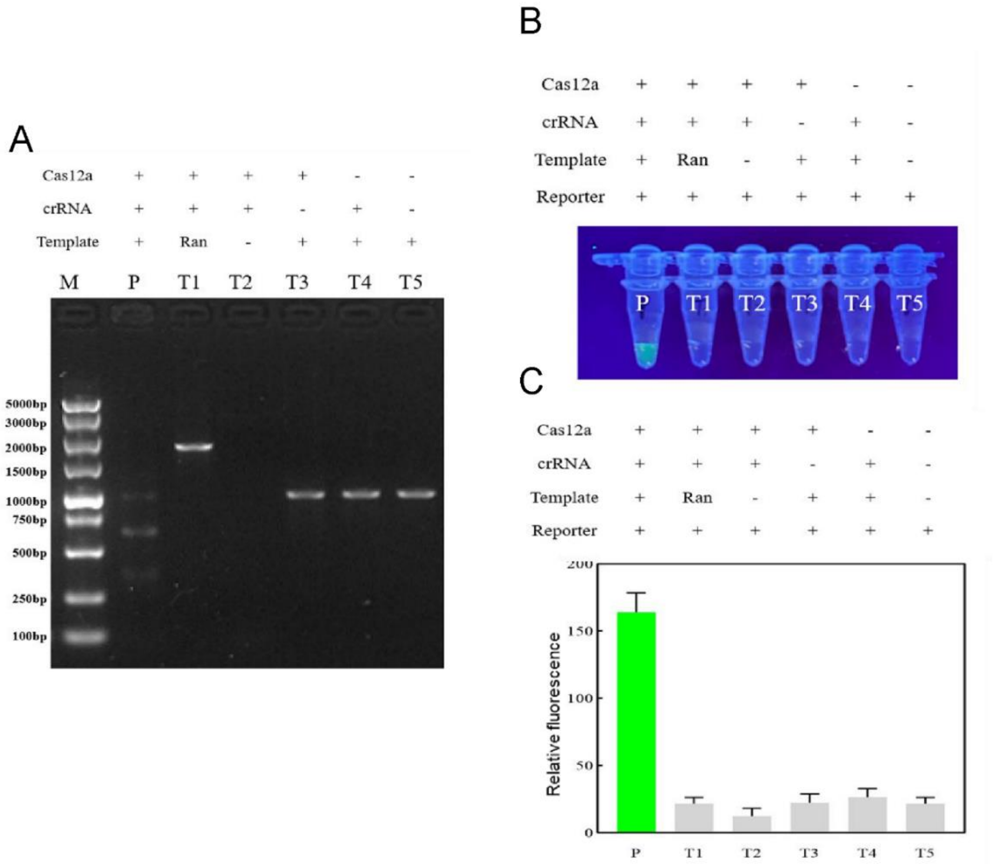

Figure S5, related to Figure 5. Optimization of conditions for LAMP and evaluation of digest activity of CRISPR/Cas12a.

A. CRISPR/Cas12a specifically digested target DNA as analyzed by agarose gel.

B. CRISPR/Cas12a specifically digested target DNA as analyzed the fluorescent signal under light.

C. CRISPR/Cas12a specifically digested target DNA as analyzed the fluorescent counter.

Table S1, related to Figure 1. List of cDNAs identified in the present study

#	Names	Sequences	Blast results (IDs/Descriptions)
**1**	>k141_1	ATAATGGAAAGAGCAAGATCCTCAAATTCAACACAGCACCGACCTAACTAAACTTGATGGTGAAACTTTGGAAGAAGTGGATACCTTTTCATACCTGGGTAGCATCATCGATGAACAAGGAGGATCCGATGCGGATGTAAGGGCAAGGATCGGCAAAGGAAGGGCAGCATTCCTACAGTTGAAAAATGTATGGGACTCCAAACAACTGTCTACCAATCTCAAAGTTAGACTCTTCAATTCGAACGTCAAGACAGTTCTTCTGTATGGAGCTGAAACGTGGAGAACTACTTCAAACATCCTCAGGAGAGTACAGGTATTTATCAACAACTGTCTACGCAGAATATTGAATGTCTGTTAGCCGTAAACCATCAGCAACAGCCTACTTTGGAAGAGAACAAACCAACTTCCAGCTGAAGAG	AY915893.1/SJCHGC09829 protein mRNA
**2**	>k141_49	GATAACTGGAGCAACAGCATTACAACGGTTCAATACAGCCTCTCTTCGGGATACTGACAAACTCAAGGAATTCAAAATAACTCTCAACAACAGGTTTCAAGCCTTACAAGATCTGCTGAACGAGGAAGAAACCACTATGGATAACAACTGGAAAAGAATCAAAGAGGCACTGACTTCAACTTGTCACGAGGCTCTGGGCTGCAAGAACTACCATCATAAGGAATAAATTTCCACAGAGACCCTGAGCAAAATTCAATAAAGGAGGAACAAGAAGGCAGAAATCAACAACAGCCGAACTAGAGCAGTGAAAATCAAGGCACAAAATGAATACACAGAAGCAAATAAGCAAGTAAAGAAGAGTATTCGAGCCGACAAACGTA	FN356221.1/non-LTR retrotransposon SjCHGCS19
**3**	>k141_56	CTGTATGGAGCTGAAACGTGGAGAACTACTGCAAACATCCTCAGGAGGGAACAATTATTTATAAACAACAGTCTACACAAGATACTCAATGTCTGTTGGCCGGAAACCATCAGCAACAGCCTACTTTGGAAGAGAGCAAACCAACTTCCAACTAAAGAGGAAATCAGGAAACGACGTTGGAAATGGATAGGACATACATCGGGGAAATCATCAGACTGAATCACGAGACAAGCACTAACTTGGAACTCCGTTTGGAAAACGGAAAAGAGGAAGGCCAAAGAACACACTGAGTCGAGAGTTGGAAGCAGACATAAAAAGGATGAACAGTAAC	AY915906.1/SJCHGC09842 protein mRNA
**4**	>k141_63	CTGTATTGATGTTTGAATTGAAGAGTCTAACTTTGAGGTTGGTTGACAGTTGTTTGAAGTCCCATATGTTCTTTAAGTGTACGAACTCCGCCTGCAGCTCTTCTAGGGTTACTGCTGGTTCCAAGCCCGGGTAAAGGAGGAGGGTTGGGCATAGGGCTAGCACCCCTATCCCGTAAAACTCAAGTTTGCTAAAAAAACGCTAACCAGAATAAACAAACCTAACCATTTAAACTCTGCCCTGGAAGTAGAAGGAACCTATATGACGCTTCATGATGAAAGCCGAGATTCTTCGGAAGTCATGAGGCCGATGCCACCCCTTCTAACGACCAGAGCAAAAATTTTTATAGATACATGGAATGTTCGGACAATGTGGTAGACCGTGAAGACCAACCAGATAGCAGCAGAAATGAAGAGATACAGCTTGACTGTGCTGGGGATCAGCGAAATGCATTGGACTGAAACTGGACAG	FN356221.1/non-LTR retrotransposon SjCHGCS19
**5**	>k141_17	ACATGTTTACATTGTCAACAATAATAGAAGATTGTTTTACTCTCTCAGTTTTATATAGCTGATAGATAATTGTATGAGATAAAGTTTACGAAGAATCAGAAGAATATTCACTGAACATGGTACTTACTTACGCCTGTTACCCCTCTCGGAGGAGCAGAGGCCGCTCACCAGTATTCTCCATTCAACTCTGTCCTGAGCAATCCTTTCCAACATTTTTCAGTTGCTGTTCATCCTTTTAATGTCTGTTTCCAGCTCTCAAAGCAGTATGTTCTTTGGCCTTCCACTTTTCCGTTTTCCAACAGGGTTCCAAGTTAGTGCTTGTCTCGTGATGTAGTCAGATGATTTCCGCAATGTATGTCCTATCCATTTTCAACG	FN356221.1/non-LTR retrotransposon SjCHGCS19
**6**	>k141_3	GTTGGAAATGGATAGGACATACATTGCGAAAATCATCAGACTGTATCACGAGACAAGCACTAACTTGGAATCCTGTTGGAAAACGGAAAAGAGGAAGGCCAAATAACACACTGCGTCAAGAACTGGAAGTAGACATAAAAAGGATGAACAGTAACTGGAGACAGCTGGAAAGGATTGCTCAGGACAGAGTTGAATGGAGAATACTGGTGAGCGGCCTATGCTCCTCCGAGAGGGGTAACAGGCGTAAGTAAGTAAGTAAGTTTAGAATAGGAAATGAAAATTGTAATGTTGATAACACTGGAAGAGAGGATAATAAGCAGTGAATAAACCTGATTATA	FN356221.1/non-LTR retrotransposon SjCHGCS19
**7**	>k141_15	CCTGTGCCCCCACTGACTGCAGTTCAGACGAGGTGAGAGATGAGTTTTACAGCAAGCTTTCGGACCTTCTCCAAAAAGCTAGGCGCTCTGATATAGTTATAATAACTGGTGACTTTAATGCTCAAGTAGGTAGACTAAGTGAAAATGAGAGACACTTGGGTGGATGTTATGGTGTCGCGGCTCAAAGAACAGATAATGGCGATCGTCTGTTGCAACTATGCTCAGATAACCGCCTGTTTCTAGCGAATACTAACTTTAGGCATAAGGAAAAACATCTTTTGACCTGGCGACCCCCAAAATCGTCTCAACGTTGGACCCAACTA	AF412215.1/non-LTR retrotransposon SjR2-like
**8**	>k141_25	GAAACTATATTAGTCAGACTGATTCCCCTGTGATTGTCACAGGAAGACTTTTGTCCTTTCTTATAAATTGGGACAATCAGGAATTGAGACCAGTCAGATGGTATTACGCCCAGTCCCCAGATTCTACCTAAGATTTCAGTCAATCTAGCTGCTAAAACTGGACCACCATCCTTAAAAATTTCAGGAGTAAGCCTATCAGGACCTGCTGCTCTACCTCGCTTCAGATTTCCTATAGCCTTCTCAACTTCATTAAGACTAGGAGGAGTTAAGTCGATTTGCCATCTAAGATGACTGGGGATCGTGGGCAACCGAAGTGTGGCTGAAGGCCAGTTGAACTGATCCCTAAAGTGTTCTGCCCATCGGTCCAATCTCCTGGACTGAGAAAGAATAATAATCCCATCCTTTTCCGAGATATTCTCACTGACATTCGGATTCC	AF412215.1/non-LTR retrotransposon SjR2-like

**9**	>k141_29	AAAACATGGCACCAAACCATGAAGTCATTGACAACTGGGCTGAGTCATGTTAATGGATGCAGACTACCTGATTGGGGCCCACGTGATGATCGTAACAATAGGTTAGAGACTC<br>TAGATGATATGGCTCAGAATCGTTCGCAATGGCGCAGGTGCATCCACTCTTTGTGTTCTCTCAAAATCTAACCTTCTGAATTCTCTATCTTTCTCCTTTCAAATTTATCTCACTG<br>AATTATACTTTTCGAATAATATCTTCGCTCCCTAATTTCTGCATTACTGCTAATACTTCTACTATCGCTATCACTCTTGGATTTGAACTGACAGTTGCGTCTCTGTACTGATGTGG<br>TATGGCGACCTGAACTGATGTACGTACGTACGAAGTTCTGCGTTGTATCTGTCTGT	AF412215.1/non-LTR retrotransposon SjR2-like
**10**	>k141_77	CCTGGGATTACTGATCTGTCCTTAATTATCATTTTGATACATTAACTGTAATCACTGTACAGATATATGTATGTATGTTATTTACAGTTAGGCTGAGCCATGTTAATAGGTGCAGAC<br>TACCTGGTAGGGGTCCGCGTGATGATCGTAACCAATGATTAGAGACTGGGTGACATGGCTCAAAATCGTTTGCAATGGCGCAGGTGCATACACTCCTTGTCTTCCTCCAAATT<br>CTAGTTTTTGAGCTTCTCATAATTTCTCTTTACTGAATCACTTTTTATTTCAAATCTTATCTCTGATCTCTATCCCTTTCCATTAGTACCTACTCTGTTACTACCTCC	AF412216.1/non-LTR retrotransposon SjR2-like
**11**	>k141_134	TGTGGCAGTGTCTGTCATTAAAAGGTGTACCAAAGAAGTACATTAACCTTATACAAGCTTTCTACTCTAAAACTACAGGTCGAGTGAGAGCTTATGGCGAACTGTCATCGGAA<br>TTCCTCACTTCAAGTGGTGTCCGTCAGGGATGTCCACTTTCCCCATTCTTGTTTAATTTTGTCATAGACATGCTCTTAGATATAACCTTATCTTCGTCTGACTTTTCAGGAGTTG<br>ACTTACTACCAGGAGCCTCGCTTACTGACTTAGAGTATGCTGATGACATAGTCCTATTTGGTGAAGACGCTGACAAAATGCAGAGTCTTCTGACTACCCTGAGCAACAATGCA<br>AGCATGTTCGGGATGCGGTTTTCCCCCTTGAAATGCAAAATGTTGCTTCAGGATTGGGTTGCATCGGCACCTGAACTAGTGATAGGGAGTGAAGTAGTCGAGCGTGTAGACT<br>GCTTCACGTATCTGGGGAGTCTCATCAGTGCTGATGGCCTGGTGACTGACGAAATCTCAGCACGGATACAGAAAGCTCGATTGGCTTTTGCCAAGTTGCGTCACTTATGGCG<br>CAGGCGAGATATCCGCTTGCTAACCAAAGGACGAGTGTACCGCGCAGCAGTTCGCTCTGTCCTACTATACGGGAGCGAAACATGGCCGTTAAGAGTAGAGGACATTCGTAGG<br>TTACTGGTCTTCGACCATAGGTGTCTCCGAAACATTGCTCGAGTATCGTGGGACAACCGAGTGAGCAATGCTGTCGTTAGGAA	AF412215.1/non-LTR retrotransposon SjR2-like
**12**	>k141_143	CAAAGTTGTAGTAACCGGTCGCCATTGTCTGTTCGTTGAGCCTCAATACCATATGATCCACCTAGGTGCCTTTCGGTTTCCTCTAGTCTACCTACTTGGGCATTAAAATCACCA<br>GCCACTATTACTACATCAGTGCGCTTTGCTTTACATCGAAGGTCATAAAGCTTTCTGTAAAATTCATCTTTCACTTCATATGAGCTGAGTGGGAGCATAGCCAGAGACAACAAA<br>GAGACAACGGCGTGTGTCCCTATCTTTACGAGTCCTTACCGTTCCATTTAGACGGACAGCGCACAAGCGACTGTCTATTAGAATCCAGTCGAGGAGAGCCTGTTCAACTTTTA<br>AACTTAGTGCTATACCTACACCAGCACGGCTTCGAGTTGTAGCAGTGGGGTCTCTGGAAACAAGAGTGAACCGAGCAGATTCCTTATTTTCACAAGGTGAGGTCAAGCGAAT<br>GACTGTGCTTGGATCCTGTATACGCGTTTCAGAGATGCAGCAGATATCGATGGAGCGGGATTCTAGAGTCATGGCTAAGGATGCCTGTTGTCCTATTTGACATAACGTTCGAAC<br>GTTGAAAGCTCCAATATACAACTTAGTGCGCGGTTTTAAGAGACCAGGGATAACGTTTTGTGAACTCAAATCACTAGCATTAGAGACGCAAGGCGATAAAATTATGTTAGAAG<br>GATTGGGCATGGTGATAAGAGAGTTATTAAGAGTTGAAGGTGGGATTTGGCTTCGAACAAGATGATCTCGTGTGCTGTTCGAGGTAATAGGGCGGTTTTCACTTCCTGGCTGC<br>TCACACCATGGAAGGG	AF412220.1/non-LTR retrotransposon SjR2-like
**13**	>k141_74	ACCTCAGAAGTACATAAACCTTGTGAAGGCTCTTTACTCGAACACTACCAGTCGAGTCAGAGCTTATGGCGAACTGTCATCTGACTTTGCGACCTCAAGTGGTGTCCGTCAA<br>GGCTGCCCACTATCTCCGTTTTTGTTTAATTTTATCATAGACGTGCTACTGGAAGTAACGCTTTCTTCGGCTGAATTTTCTGGGATTGATCTCCTTCCAGGAGGTCCACTCATCG<br>ACTTAGAATACGCAGACGACATAGTCCTGTTTGGTGAAGACACTGATAAGATGTAGTCTTCTGGTCGCACTGAGCAACAATGCCAGGATGTTTGGGATGCGTTTCTCCCCCTC<br>TAAGTGTAAATTTTTACTTCAGGACTGGCCTGCGTCAACCCCTGAACTAAGGATAGGGAG	FN356222.1/retrotransposon SjCHGCS20
**14**	>k141_90	GTACTCTCCACGAGAGTCGGACCATCTAGACTATATCTCTCGATTCTCTACGGACATACGACATATATCGGGAGCTAACAATATAGTTGCTGACGCCTTATCGAGAATACATTCC<br>TTGAATCGTTTCCTAGGAATCGACCTTGTTAAACTAGCTCAACTTCAAAGTGAAAATATCGATTTTCATCACGAGTTAGCCGCAACAACGCTCCAACTCCAAACTAAAACGAT<br>TGGAAAAGGTAAGAACACTTTAATTTGTGATTCTACAGGTATAGCTTGTCCAGTCGTCTCCAGGAGTTATCGACTTGTCATTTTCGACACATTACACA	FN356203.1/LTR retrotransposon SjCHGCS1
**15**	>k141_125	AGGTACATCCAGCCGACGAGTCCCAAATAGGACGAAACGCGCGTCCTGGATTCCACTTGCCAGTCACAATCCAACTTAAATATAACTTGTGTAATACCGCTATATAGAGGCAA<br>TCCGCTCAGGATGCACATATGGCAACAGAGGCTGACCAGTTTCAGTCCATTACAAATCAACGGGAAGATACAAGCCTATTCTGAATTAGATATGTACCCGTTGCACAAGTGTG<br>ACTGTCTGGACACAGTAGCCTAGTGGTGAACGCGGTGGCGTTTGACACGAAGCGGACTGAGTTCGAATCCCAGTGTGAACATCAACATTGCGATGCAGGTACATCCAGCCA<br>ATGAGTCCCAAATAGGACGAAACGCGCGTCCTGGATTCCACTTGCCAGTCACAATCCAACTTAAATA	AF213692.1/Sj-alpha-1 retroposon-like
**16**	>k141_35	CAGCAGGTGCCTACCTTTTATAATGGGGTGGGGATGTGTAGACGTTCTTATACGGCGCACGCATTGGAGGTGTGAGGGTCTGCTTGTCAGTGCACTTTCTCAGAGTATTCACC<br>ACGACCGGCATTGCTGCCTTTCTACTATGGCCAAACTGATCAGGTTTGACTATTATTGTTGAGCTTGGTTGGGTCGGCAGGTGACTCTTTGGTTCGTAATTAATTATTATGGACC<br>ATGAGTGTTACTAACCGGCTGTAGTGGAATTGTGTAGTGTGTCGGAGATGGCGGCTTCACGTGCGTGCTTTGTCCTTCGGG	Z46504.4/*Schistosoma japonicum* 28S rRNA gene

**17**	>k141_39	GACGGTAGAGGTGGATTAGACACACATTGAGGAAACCACCAAATTGTGTCACAAGACAAGCACTAACATGGAACCCTGAAGGTCAAAAGAAAAGAGGAAAACTAAAGAACATACACTATTGGGAATCGGAGACAGACATGAAGAAAATGAAGAGCGTTTGGGAAGGGCTGGAAAGGGAAGCTCGGGACAGAGTTGGTTGGAAAATGCTGGTTGGCGGCCTATGTTCTATTTGAGGCAACAGGCATTGATTGATTGATTGATTAAAATTTATTACCATAAAATCAAGTCATTTTGTAGATCCACATTGATTCAACTCAAAGTAGAAGTTTATCCTTCAC	/clone BAC C108_07O23
**18**	>k141_50	TCTTTATATGGGGAAGAAGGTTGTGCACGACATTCACCCCTATTTCACGATTCATGCACAACTCATCACCGCCAATCAACTTACACCCACAACATCTCGCCATCATCTTTATATGGGGAAGAAGGTTGTGCACGACATTCACCCCTATTTCACGATTCATGCACAACTCATCACCGCCAATCAACTTACACCCACAACATCTCGCCATCATCTTTATATGGGGAAGAAGGTTGTGCACGACATTCA	AY812939.1/SJCHGC08404 protein mRNA
**19**	>k141_54	ACTTTATATGGGGAAGAAGGTGGTGCACGACATTCCTACCAATTTTTTAATTCACAACTCATCACCGCCAATCAACTTACACCCACAGCATCTCGCCATCAACTTTATATGGGGAAGAAGGTGGTGCACGACATTCCTACCAATTTTTTAATTCACAACTCATCACCGCCAATCAACTTACACCCACAGCATCTCGCCATCAACTTTATATGGGGAAGAAGGTGGTGCACGACATTCCTACC	AY812939.1/SJCHGC08404 protein mRNA
**20**	>k141_59	GGGGGCAATGTCGTGCACAACCTTCTTCCCCATTTAAAGATGATGGCGAGATGTTGTGGGTGTAAGTTGATTGGCGGTGATGAGTTGTGGATGAAAAAATAGGGGGCAATGTCGTGCACAACCTTCTTCCCCATTTAAAGATGATGGCGAGATGTTGTGGGTGTAAGTTGATTGGCGGTGATGAGTTGTGGATGAAAAAATAGGGGGCAATGTCGTGCACAACCTTCTTCCCCATTTAAAGA	AY812939.1/SJCHGC08404 protein mRNA
**21**	>k141_67	ATTGTGACTGGCAAGTGGAATCCAGGACGCGCGTTTCGTCCTATTTGGGACTCGTCGGCTGGATGTACCTTCATCCCAATGTTGATGTTCACAGTGGGATTCGAACTCAGTCCGGTTCGTGTCATACGCCACCGCGTACACCACTAGACTACTGAGTCCAGACAGTCACGTTTGTGCAATGGGCGAATTGGTAGTAGAATGAATTTGTATATTCCCATTGATTTGTTAAAGACTGAAGCTGGTCAGTCACTGTTGCTATATGTGCATCCTGAGCGGATTGCCTCGATATAGCGATTATTCACAAGTATTATTTTTAATTTGTATTGTAACTGGCAAGTGGAATCTAGGA	FN293031.1/clone BAC C108_75H07
**22**	>k141_5	ACAGAAATAAATTGGTTATTCAAGTTAAATAACTTAAAATCCTTTTGGTTATGTTAGTGGAAAAGTTGTTTTAGCACGCATAGTTCTTGTTCATCTATATGCGCAGAAACAGTTTTAGCAACGGGCTCATAGGTAATTAGGATGAACTTGTATCTTCCCGTTGGTTTGTTATGGACAACAACTGGTCAGTCTCTAGTATATGAACATCAACATTGGAATGCAGGTACATCCAGCCGACGAGTCCCGAATAAGACGAAACGCGTCTTCTGGATTCCCATTGCTAGTAACAATTCAACTTAGAGTAGACATGTTCTAACGCTTACT	AY915795.1/SJCHGC09721 protein mRNA

Table S2, related to Figure 2A. The list of primers used in the present study for PCR amplification

Names	Primers	Sequences (5'-3')	Amplified sequences/genes
Actb	Forward	GCACCACACCTTCTACAATGA	NM_007393.5
	Reverse	CACGGTTGGCCTTAGGGTTC	
k141_1	Forward	ATGGTGAAACTTTGGAAGAAGTG	k141_1
	Reverse	CTTACATCCGCATCGGATCCTC	
k141_49	Forward	CCTCTCTTCGGGATACTGACAA	k141_49
	Reverse	CATAGTGGTTTCTTCCTCGTTCA	
k141_63	Forward	CATGATGAAAGCCGAGATTCTTC	k141_63
	Reverse	TGATCCCCAGCACAGTCAA	
k141_56	Forward	ACCAACTTCCAACTAAAGAGGAAATC	k141_56
	Reverse	GTCTCGTGATTCAGTCTGATGATTT	
k141_17	Forward	CCGCTCACCAGTATTCTCCATT	k141_17
	Reverse	AAAAGTGGAAGGCCAAAGAACA	
k141_3	Forward	ATAACACACTGCGTCAAGAACT	k141_3
	Reverse	ATTCAACTCTGTCCTGAGCAATC	
k141_15	Forward	GAGTTTTACAGCAAGCTTTCGGA	k141_15
	Reverse	CATCCACCCAAGTGTCTCTCATT	
k141_25	Forward	CAGACTGATTCCCCTGTGATTG	k141_25
	Reverse	AGTTTTAGCAGCTAGATTGACTGAAAT	
k141_29	Forward	GGTCCGCGAGACGAAAGTCACCAATGGTTA	AF412215.1
	Reverse	TGCAGAAAATTAGGGAGCGAAGATATGATTTGA	
k141_77	Forward	AGGTGATATGGCTCAAAATCGATT	AF412216.1
	Reverse	GGAGGTAGTAACAGAGTAAATACTAACGGAAAG	
k141_134	Forward	GGTCGAGTGAGAGCTTATGGC	AF412215.1
	Reverse	TCCTGAAAAGTCAGACGAAGACA	
k141_143	Forward	AGCGAATGACTGTGCTTGGAT	k141_143
	Reverse	GTTGTATATTGGAGCTTTCAACGTTC	
k141_74	Forward	TGCGATCTCAAGTGGTGTCC	FN356222.1
	Reverse	CGTCTGCGTATTCTAAGTCGATG	
k141_90	Forward	TGAAAATATCGATTTTCATCACGAG	k141_90
	Reverse	GACGACTGGACAAGCTATACCTGTAG	
k141_39	Forward	AAACCACCAAATTGTGTCACAA	k141_39
	Reverse	TCCCTTTCCAGCCCTTCCCAA	
k141_35	Forward	ATGTGTAGACGTTCTTATACGGCG	k141_35
	Reverse	ACCTGATCAGTTTGGCCATAGTAG	
k141_125	Forward	AGTCCATTACAAATCAACGGGAA	k141_125
	Reverse	GTTCACCACTAGGCTACTGTGTCC	
k141_67	Forward	CGTTTCGTCCTATTTGGGA	k141_67
	Reverse	CTCAGTAGTCTAGTGGTGTACGCG	
k141_5	Forward	GCACGCATAGTTCTTGTTCATC	k141_5
	Reverse	GACTGACCAGTTGTTGTCCATAAC	
k141_50	Forward	GAAGAAGGTTGTGCACGACATT	k141_50
	Reverse	AAAGATGATGGCGAGATGTTGT	
k141_54	Forward	GAAGAAGGTTGTGCACGACATT	AY812939.1
	Reverse	GTAAGTTGATTGGCGGTGATGA	
k141_59	Forward	GCACAACCTTCTTCCCCATTTA	k141_59
	Reverse	ACAACATCTCGCCATCATCTTT	

Table S3, related to Figure 2B. The list of qPCR primers for cDNAs

Names	Primers	Sequences (5'-3')	Amplified sequences/genes
k141_1	Forward	ATGGTGAAACTTTGGAAGAAGTG	k141_1
	Reverse	CTTACATCCGCATCGGATCCTC	
k141_49	Forward	CCTCTCTTCGGGATACTGACAA	k141_49
	Reverse	CATAGTGGTTTCTTCCTCGTTCA	
k141_63	Forward	CATGATGAAAGCCGAGATTCTTC	k141_63
	Reverse	TGATCCCCAGCACAGTCAA	
k141_15	Forward	GAGTTTTACAGCAAGCTTTCGGA	k141_15
	Reverse	CATCCACCCAAGTGTCTCTCATT	
k141_25	Forward	CAGACTGATTCCCCTGTGATTG	k141_25
	Reverse	AGTTTTAGCAGCTAGATTGACTGAAAT	
k141_29	Forward	GGTCCGCGAGACGAAAGTCACCAATGGTTA	AF412215.1
	Reverse	TGCAGAAAATTAGGGAGCGAAGATATGATTTGA	
k141_77	Forward	AGGTGATATGGCTCAAAATCGATT	AF412216.1
	Reverse	GGAGGTAGTAACAGAGTAAATACTAACGGAAAG	
k141_134	Forward	GGTCGAGTGAGAGCTTATGGC	AF412215.1
	Reverse	TCCTGAAAAGTCAGACGAAGACA	
k141_74	Forward	TGCGATCTCAAGTGGTGTCC	FN356222.1
	Reverse	CGTCTGCGTATTCTAAGTCGATG	
k141_90	Forward	TGAAAATATCGATTTTCATCACGAG	k141_90
	Reverse	GACGACTGGACAAGCTATACCTGTAG	
k141_39	Forward	AAACCACCAAATTGTGTCACAA	k141_39
	Reverse	TCCCTTTCCAGCCCTTCCCAA	
Actb	Forward	GCACCACACCTTCTACAATGA	NM_007393.5
	Reverse	CACGGTTGGCCTTAGGGTTC	
Spiked in control	Forward	GGCGCTGGTGCATATGTAGAAGTT	/
	Reverse	GCATACATGTAAAGCATGCAGACGT	

Table S4, related to Figure 5. The list of primers used for LAMP (For crRNA-F and crRNA-R, the underlines indicate T7 promoter, bold indicates the repeated sequences binding to Cas12a, box indicates PAM sequence)

**Primer names**	**Sequences (5'-3')**
R2LS-F	GGATCCCGTGGGACAACCGAGTGAGC
R2LS-R	GAATTCAACACAAAGAGTGGATGCACC
SJ-R2LS-F3	GCTTCATCAGTTGAGGTGG
SJ-R2LS-B3	TTGGTGACTTTCGTCTCG
SJ-R2LS-FIP	TCCAACCTATTCCTACGCCGTTACGCATATCTAACTACCGTC
SJ-R2LS-BIP	GGCGGCCAAACCAAAACATGCCTACATCTACCAACATGACT
SJ-R2LS-LF	AACATAGCTCGTCGAGGCA
SJ-R2LS-LB	GCAGAAATCGATGAAGTCACTGA
crRNA-F	GAAATTAATACGACTCACTATAGG**GTAATTTCTACTAAGTAGAT**AAGTCGGAAACAATAAAC
crRNA-R	GTTTATTGTTTCCGACTT**ATCTACTTAGTAGAAATTAC**CCTATAGTGAGTCGTATTAATTTC

Table S5, related to Figure 6. The list of primers and probe used in the present study for LF-RPA

Primer names	Sequences (5'-3')
R2LS-F(LF-RPA)	GGTCCGCGAGACGAAAGTCACCAATGGTTA
R2LS-R(LF-RPA)	biotin- TGCAGAAAATTAGGGAGCGAAGATATGATTTGA
R2LS-probe	FAM-GGCGCAGGTGCATCCACTCTTTGTGTTCTC [THF]CAAAATCTAACCTTC-C3 spacer
